# Supplementary material for: Hybrid-DIA: intelligent data acquisition integrates targeted and discovery proteomics to analyze phospho-signaling in single spheroids
Source: Nat Commun. 2023 Jun 16;14:3599. doi: 10.1038/s41467-023-39347-y (PMC10276052; doi:10.1038/s41467-023-39347-y)
Supplement: Supplementary file 1 — Supplementary information [file 41467_2023_39347_MOESM1_ESM.pdf]

Supplementary Material for:

Hybrid-DIA: Intelligent Data Acquisition Integrates Targeted and Discovery Proteomics to analyze Phospho-signaling in Single Spheroids

Ana Martínez-Val[1], Kyle Fort[2], Claire Koenig[1], Leander Van der Hoeven[1], Giulia Franciosa[1], Thomas Moehring[2] Yasushi Ishihama[3], Yu-ju Chen[4], Alexander Makarov[2], Yue Xuan[2], Jesper V. Olsen[1]

[1] Novo Nordisk Foundation Center for Protein Research, University of Copenhagen, DENMARK

[2] Thermo Fisher Scientific, Bremen, GERMANY

[3] Kyoto University, Kyoto, JAPAN

[4] Academia Sinica, Taipei, TAIWAN

*Correspondence to: jesper.olsen@cpr.ku.dk, yue.xuan@thermofisher.com.

- Supplementary Note 1: instructions to use the hybrid-DIA API in and Exploris 480 MS.
- Supplementary Note 2: guide for post-acquisition processing analysis of targeted scans from hybrid-DIA runs.
- Supplementary Figures 1-6

Supplementary Note 1. Instructions to use the hybrid-DIA API in and Exploris 480 MS.

In order to use hybrid-DIA acquisition in Exploris 480, follow these steps.

1. Download the folder "Exploris" from https://github.com/thermofisherlsms/MoonshotApps onto your local computer. Make sure the following items are present: Moonshot_V1_4.exe Thermo.API.Exploris-1.0.dll, Thermo.API.Spectrum-1.0.dll and Thermo.API-2.0.dll.
2. Ensure that your Exploris Instrument has an API license.
3. Double click the application "Moonshot_V1.4.exe" within the "Exploris" folder. The GUI shown in Figure 1 should open and the console message box shown in Figure 2.

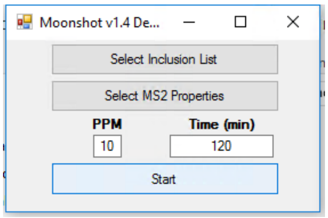

Figure 1. Main screen of the Moonshot API Interface

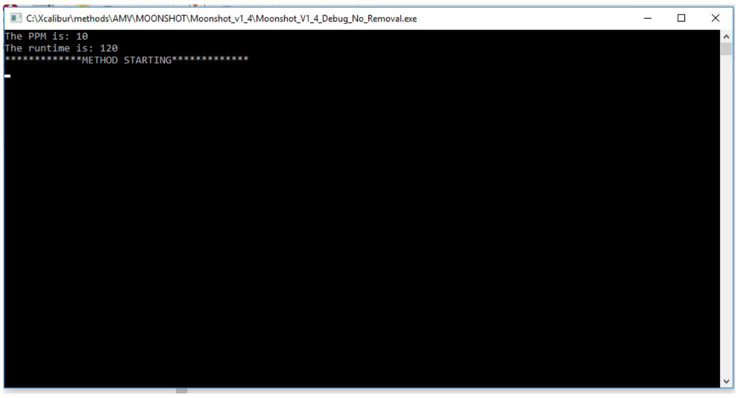

Figure 2. Secondary console window. Messages from the program will be displayed here.

4. Press "Select Inclusion List" and load a properly formatted inclusion list (see Figure 3 and section 4.1 for formatting on the inclusion list). The message "Inclusion loading was successful!" should appear in the console message box.

	A	B	C	D	E	F	G	H	I	J
1	Parent Ion	Parent Charge	Retention Time Low	Retention Time High	Triggered Mass	Parent Ion Fragments				
2	577.8103	2	2.7	3.2	400.7014	239.1134	568.8055	333.2126	916.5084	
3	488.7267	2	2.7	3.2	616.8133	701.3192	630.2824	573.2615	458.2348	343.2081
4	381.8759	3	3.3	3.7	497.2526	902.472	773.4288	674.3607	551.3183	511.2979
5	403.2339	3	3.6	4.2	604.3472	908.5052	851.1824	752.4147	681.3772	496.2991
6	654.3091	3	4.1	4.5	981.4625	1159.5387	1088.5023	932.4495	888.3864	559.2279
7	513.3074	2	4.5	4.8	750.3401	912.5264	855.5032	685.3976	628.376	428.261
8	488.2779	2	4.8	5.2	356.5443	862.4655	765.413	652.3288	553.2976	431.7366
9	454.2646	2	5.3	5.8	670.6352	836.4861	765.449	666.3805	565.3337	365.2178
10	737.9198	2	5.3	5.8	1005.4503	1276.731	1163.6508	1064.583	879.5031	582.3345
11	713.364	2	7.5	7.7	543.3013	1149.5768	1062.5457	965.4925	850.4667	531.7762
12	895.9474	2	7.5	7.8	959.5194	1298.6666	1201.6143	1086.5873	901.5084	802.4395
13	1204.0403	2	7.8	8.2	1284.0854	1718.8663	893.4712	822.434	652.3276	537.3022
14	1004.4476	2	7.8	8.2	879.4848	1216.5499	386.1374	499.2212	702.3935	580.2673
15	919.3987	2	7.8	8.2	810.3566	1337.6163	1266.5803	1195.5436	983.3909	884.3224
16	803.0292	3	7.8	8.2	697.3751	1440.7379	1341.605	1142.6755	442.2762	626.3972
17	731.9089	2	7.8	8.2	637.8643	1150.5867	1053.5349	956.4828	784.3965	575.7966

Figure 3. Example of a properly formatted inclusion list that is uploaded to the Moonshot Workflow by using the "Selection Inclusion List" button from Figure 1

4.1. Inclusion List Columns and formatting: the headers "Parent Ion, Parent Charge, Retention Time Low, Retention Time High, Triggered Mass, Parent Ion Fragments" must be included. File should be saved as a tab-limited text file. Other delimiter, e.g. commas, are not supported. Each row represents a targeted ion.

- **1: Parent Ion.** The precursor mass list. These precursor values should be the mass of the internal standard peptides that have been added to the sample. If one of these parent ions is present within an MS1 scan (within the retention time window, see below), it will trigger a customized MS2 scan with isolation width, NCE, and first mass as defined by the MS2 properties. If more than one parent ion is present in an MS1, a multiplexed (MSx) MS2 scan will be sent with all present parent ions co-isolated and co-fragmented

- **2: Parent Charge.** The charge state of the parent ion. For a customized MS2 scan to successfully be triggered, the parent ion's charge state must match the charge state outlined in this column.

- **3: Retention Time Low.** The lower boundary of the retention time search for this specific parent ion. An ion present in an MS1 scan that has the parent ion mass (column 1) and parent charge (column 2), but that occurs before this retention time will not trigger a customized MS2 scan.

- **4: Retention Time High.** The upper boundary of the retention time search for this specific parent ion. An ion present in an MS1 scan that has the parent ion mass (column 1) and parent charge (column 2), but that occurs after this retention time will not trigger a customized MS2 scan.

- **5: Triggered Mass.** The triggered mass associated with the parent ion. Upon triggering a customized MS2 scan for the parent ion, this scan is analyzed for the known fragments (see Parent Ion Fragment Ions below). If the customized MS2 scan shows the required number of matched fragment ions, it will trigger another customized scan whereby the parent ion and the trigged mass are co-isolated and co-fragmented.

- **6: Parent Ion Fragment Ions.** The known fragments of the parent ion. These fragment masses will be utilized when the Moonshot Workflow analyzes customized MS2 scan of the parent ion/s. This field can have multiple columns associated with it where the number of known fragment ions is not limited. Each fragment ion should have its own column.

5. Press “Select MS2 Properties” (Figure 4). Set the “Defined First Mass”, “NCE”, and “Isolation Width”, “AGC Target”, “Max IT (ms)”, “MS Trigger Intensity Threshold” and “Dynamic Exclusion (s)” as per the requirements of your experiment. Press “Update MS2 Properties”.

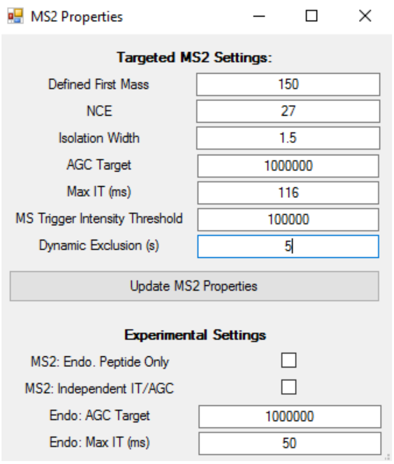

Figure 4. MS2 settings screen that appears after clicking on Select MS2 Properties in the main screen (Figure 1)

6. Modify the “PPM” and “Time (min)” in the main API screen (Figure 1) as per the requirements of your experiment, if needed. “Time (min)” should exceed the entire queue length, including loading steps. ‘PPM’ is the mass tolerance setting applied for the parent ions, triggered mass, and parent ion fragment ions in the inclusion list.

7. Start LC Queue, and immediately afterwards press Start on the API.

8. Once the acquisition of the raw file starts, the API will start to monitor the targets (parent ions) in the inclusion list. When a precursor mz in the inclusion list is found in the corresponding time frame and above the MS intensity threshold, the console output will the updated. Figure 5 shows an example of console output.

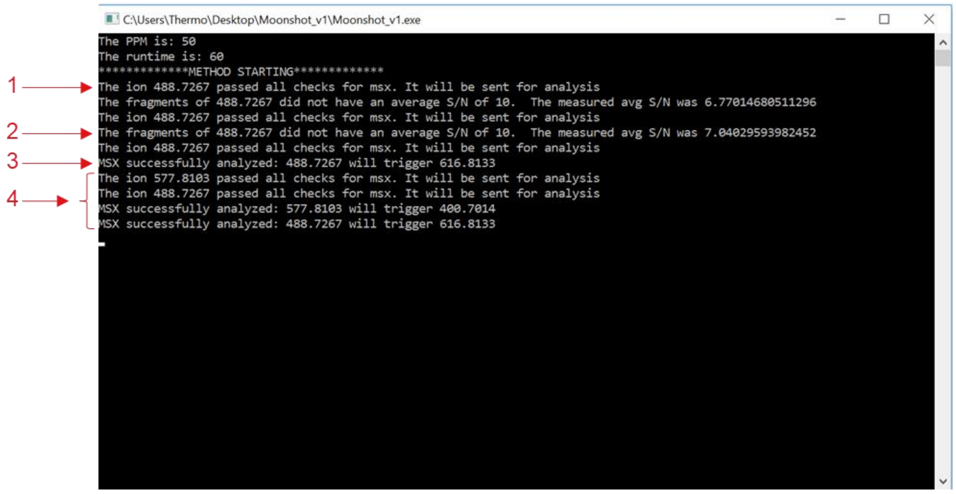

Figure 5. Secondary console window showing messages during MS acquisition in a hybrid-DIA run.

#1: The message indicates that the parent ion 488.7267 was present with the retention time window defined by the lower and upper retention boundary conditions in the inclusion table, i.e. Retention Time Low and Retention Time High, respectively. This mass will then be isolated with a narrow isolation window (as defined by the Isolation Width of the MS2 Properties) and fragmented in a customized MS2 scan.

Scans: *Full MS1 → MS2 of 488.7267*

#2: The message indicates that the customized scan of 488.7267, which was generated by Example 1, showed some known fragment ions (see "Parent Ion Fragment Ions" in section Format of Inclusion List); however, the average signal-to-noise (S/N) was not high enough (>10) to trigger an MSX MS2 with the parent and triggered masses.

Scans: Full MS1 → MS2 of 488.7267 → Analysis failed so: Continuation of normal method scans (e.g. DIA Windows, Full Scan etc.)

#3: The message indicates that a customized scan of 488.7267 (a different customized scan than generated in Example 1 and analyzed in Example 2) was successfully analyzed, meaning the known fragment ions were present at a S/N of >10, therefore a MSX where the parent ion, 488.7267, and the triggered mass (see "Triggered Mass" in section Format of Inclusion List) 616.8133 will be co-isolated and co-fragmented

Scans: Full MS1 → MS2 of 488.7267 → Analysis passed so: MS2 MSX of 488.7267 and 616.8133 → Continuation of normal method scans (e.g. DIA Windows, Full Scan etc.)

#4: This message block shows the entire workflow message output when more than one Parent Ion is present within an MS1 scan. Both 577.8103 and 488.7267 were present in the MS1. An MSX where both of these masses were co-isolated and co-fragmented was generated. Analysis of this MSX scan showed the fragments of both parent ion present; therefore, two customized scans are sent. The first is an MSX scan that co-isolated 577.8103 and its corresponding triggered mass of 400.7014. The second is an MSX scan that co-isolated 488.7267 and its corresponding triggered mass of 616.8133.

Scans: Full MS1 → MS2 MSX of 577.8103 and 488.7267 → Analysis passed so: MS2 MSX of 577.8103 and 400.7014 → Analysis passed so: MS2 MSX of 488.7267 and 616.8133 → Continuation of normal method scans (e.g. DIA Windows, Full Scan etc.)

9. Two files will be generated during hybrid-DIA acquisition and they are located in "C:\ProgramData\Thermo\Exploris\Instrument\Moonshot"
   - 9.1. A log-file containing the data from the API acquisition (e.g. 2022_12_14_10_15 Moonshot Workflow.txt).
   - 9.2. An accessory file with all information from each full scan (Scan number, mz, charge, intensity and resolution). This file can be used for troubleshooting purposes. It is named with a time-stamp followed by the name of the corresponding raw file. These files can be deleted if the space in the disk is limited.

Supplementary Note 2. Guide for multiplex IS/ENDO extraction and data analysis is hybrid-DIA files.

Extraction of MSx scans: MSx_Extractor.py[1]

Software required:
Python version: 3.9.7 (https://www.python.org/downloads/release/python-397/), Proteowizard version 3.0.21246 (https://proteowizard.sourceforge.io/)
Python Packages required: tkinter, sys, time, os, pymsfilereader, pathlib[1]

<u>INPUT</u>:
- Dir: folder with raw files to convert.
- IS-ENDO file: pairs of mz for IS and ENDO peptides (Figure 1)

```
434.881286,432.209886
1009.486558,1004.482423
1044.976839,1040.969739
1048.145059,1045.473659
1189.560505,1186.224415
1206.039222,1201.035088
367.931562,365.429495
408.211336,404.204237
409.203661,405.867572
424.722068,420.714969
448.194502,444.187402
```

Figure 1. Example of IS-ENDO File

<u>OUTPUT</u>:
- Mzml files: one mzml file per raw file containing only the desired MSx scans. To be used as input for Skyline.
- Results.txt files: one per raw file, contains the injection times per scan. Required for IT normalization. Copy all txt files into a new folder and label it as "txt".
- Config.txt: one file per raw file, used internally by ProteoWizard. They can be deleted when the conversion is finished.

<u>BEFORE START:</u>
1. Locate the folder that contains the installation of ProteoWizard.
2. Open with a text editor (such as NotePad++) the app "MSx_Extractor.py"
3. Go to line 62 in the python script and update the directory to the one where you have installed ProteoWizard. Remember to keep the "\\" between subdirectories.
4. Copy all the raw files into a new folder.

<u>RUNNING THE APP:</u>
1. Double click on MSx_Extrator.py. Two windows would appear (Figure 2 and 3), one corresponding to the GUI and the python console.
2. Upload IS ENDO file by clicking on "Open file" and navigating to the location of the file.
3. Indicate the folder that contain the .raw files of interesting by clicking on "Select Dir".
4. Click "Go!" to start the analysis. A message saying "Analyzing sample n/n" would appear and it would be updated every time a raw file is done. When all raw files have been processed, the message "Done" will appear.
5. During the raw file processing the python console (Figure 3) would show ProteoWizard actions.

[1] For help installing python packages: https://packaging.python.org/en/latest/tutorials/installing-packages/#id18

6. When done, three files would have been generated per raw file: .mzml, results.txt and config.txt. For convenience in further steps, copy all results.txt files into a new folder.

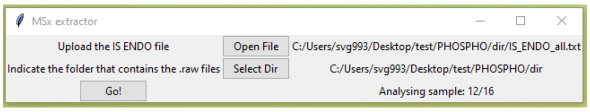

Figure 2. MSx_Extractor.py main window.

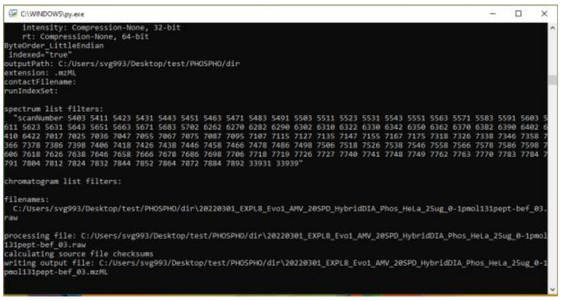

Figure 3. Python console.

Import into Skyline: Extraction of the transitions.

Required: Skyline template with the desired transitions (.sky) and fragment library (.blib).

1. Edit the following parameters in the "Transition settings tab" (see figure below for details):
    - Filter tab: remove "b" ions from ion types. They cannot be used for quantification since they are shared between the heavy and light peptides.
    - Instrument tab: set "method match tolerance m/z" to 0.001.
    - Full-Scan:
        - MS1 filtering: none
        - MS2 filtering: PRM

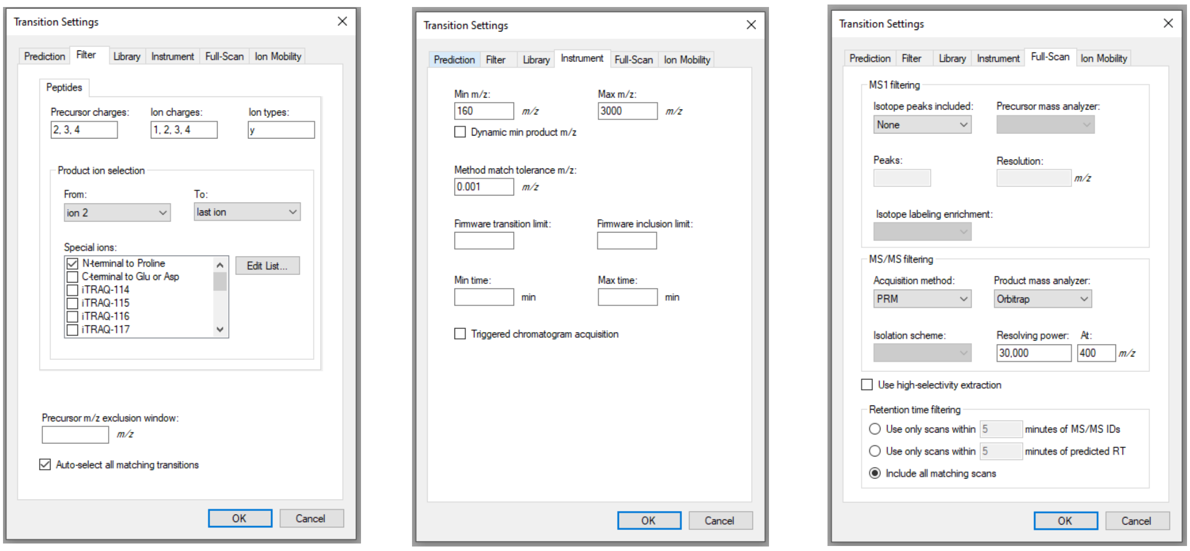

Figure 4. Transition settings tab in Skyline with specific parameters required for the succesfull import of the processed mzml files.

2. Import mzml files: File > Import > Results
3. Open Document grid (Alt+3 or View>Document Grid). Edit a document to contain the following information and save the report as "Quantification_IS_ENDO_forHybrid".
    a. Peptide.
    b. Protein. IMPORTANT: This header would indicate in the next step the IDs for each IS/ENDO pair. In the case of more than one peptide per protein, modify the header to "ProteinName", and rename "Peptide" column to "Protein".
    c. Replicate.
    d. Raw Intensities.
    e. Raw Times.
    f. Fragment Ion.
    g. Product Mz.
    h. Isotope Label Type.
    i. Mass Error PPM.
    j. Peptide Peak Found Ratio.
    k. Raw Spectrum Ids.
4. Export Report "Quantification_IS_ENDO_forHybrid" (.csv format).

5. Open Document grid again and load the table named “All_Precursors_Initial_Survey”. Edit the table to contain only two columns:
    a. Protein Name. IMPORTANT: This header would indicate in the next step the IDs for each IS/ENDO pair. In the case of more than one peptide per protein, choose the appropriate column for each unique pair (i.e: Modified Peptide Sequence). The content needs to match the content of the “Protein” column from the Quantification report.
    b. Precursor Mz.
6. Export Report “All_Precursors_Initial_Survey” (.csv format).

Injection time normalization, quantification and visualization of results.

Software required: R (v4.0)
R packages required: shiny, shinyFiles, shinycssloaders, dplyr, ggplot2, data.table, gridExtra, tidyr, ggpubr, MESS, config

1. Copy the file app.R and config.yml to your computer into a folder called "Shiny-APP". The file "config.yml" contains certain parameters to filter the data do the quantification. There data in the file can be used as a default, but it can be modified if needed. The parameters are:

    ppm: max error mass allowed. Default: 10 ppm
    n_y: min number of y fragments required for quantification. Default: 1 (recommended: 3)
    ppfr: peptide peak found ratio from Skyline. Default: 0.5

2. Open R (it can be the R console or Rstudio) and type:

```
library(shiny)
setwd("C:/Users/xxxx/xxxx/dir_shiny_app/")
#Indicate the location of the folder "Shini-APP"
runApp("Shiny-APP")
```

   or click on "Run App" on the right top corner.

3. A shinyApp will open in a new window (Figure 5 and 6).
   Input:

    (1) directory where the txt files generated in the first step are stored.
    (2) common pattern in the raw files. If the raw files share a common pattern and it has been removed during Skyline processing, indicate it here.
    (3) Maximum injection time (IT) used during hybridDIA acquisition
    (4) Precursor MZ list: csv file that contains the mz for the heavy and light peptides together with their id (phospho-site, peptide).
    (5) Quantification results: csv files exported from Skyline in the previous step. When this document is loaded, two selection menus will appear, one for the sample and another for the peptide/phospho-site.

   Output:

    (6) Plot phospho-site: this button will plot the XIC (6a) of the IS and ENDO peptide for the selected phospho-site in the selected sample before and after IT normalization. Also, it will show a profile plot (6b) of the relative intensity (calculate from the ratio IS/ENDO) of that peptide across samples.
    (7) Print Heatmap: this button will plot the relative intensity (calculates from the ratio IS/ENDO) of all peptide across samples in the form of a heatmap. (In the "All results" tab).
    (8) AUC IS, AUC ENDO: these buttons will export a table with the AUC for the heavy (IS) (8b) and light (ENDO) (8a) counterparts for all peptides, normalized by IT.

(9) If this check-box is active, the profile plot will show the ratio IS/ENDO (heavy to light) and not the scaled values across replicates. Also, the heatmap will be plotted using the ratio IS/ENDO.

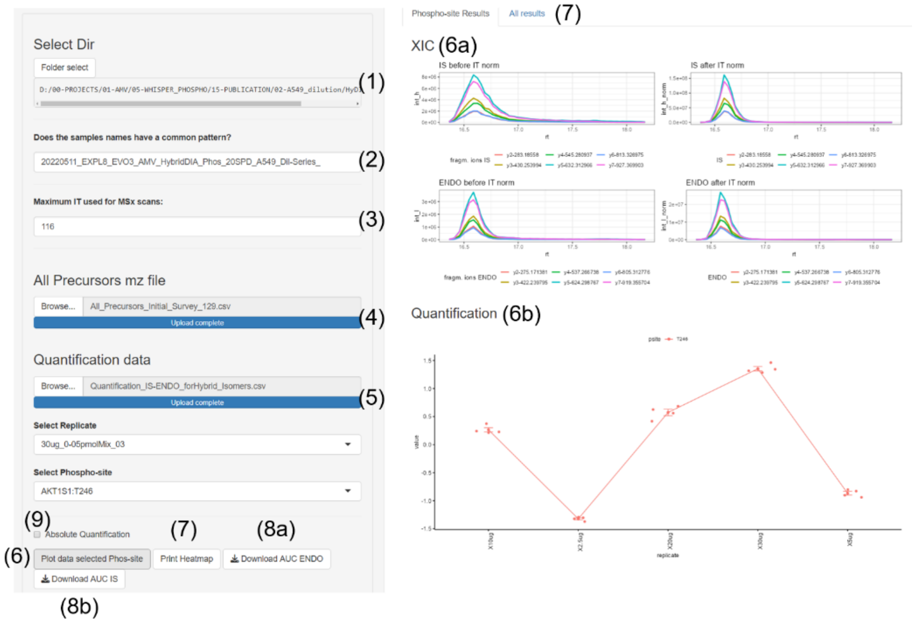

Figure 5. Shiny App interface. On the left panel: option menu to upload required files. Once Quantification data file is uploaded the selection tabs "Selection Replicate" and "Select phospho-site" appears for the user to choose what to plot. On the right panel: (top) XIC of peptide AKT1S1-T246 for sample "30ug, replicate 3", (bottom) relative quantification of that peptide among the samples compared.

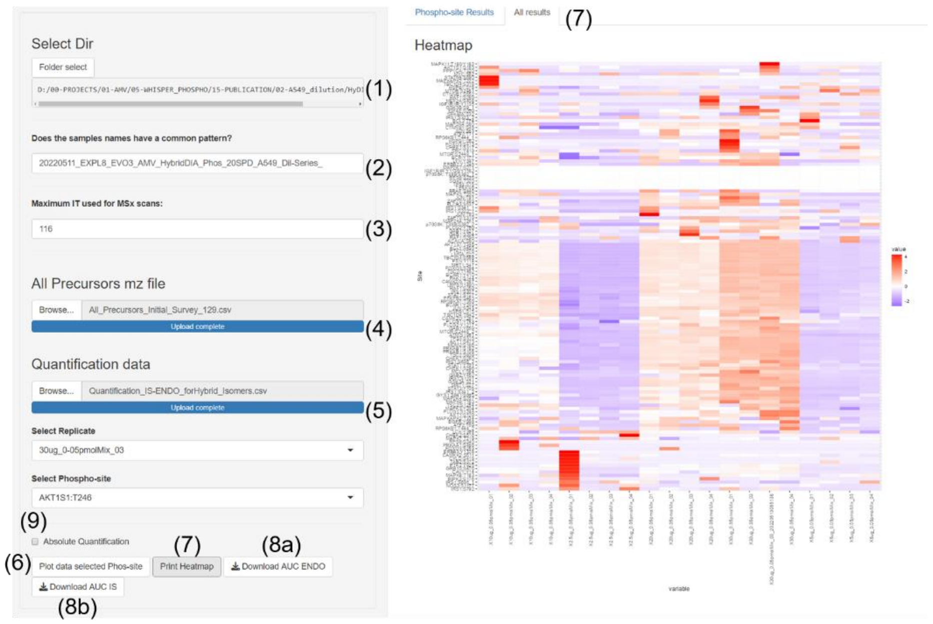

Figure 6. Shiny App Interface. On the right panel: Heatmap with the relative quantification across samples of all peptides monitored in the loaded hybrid-DIA experiment.

Supplementary Figure 1.

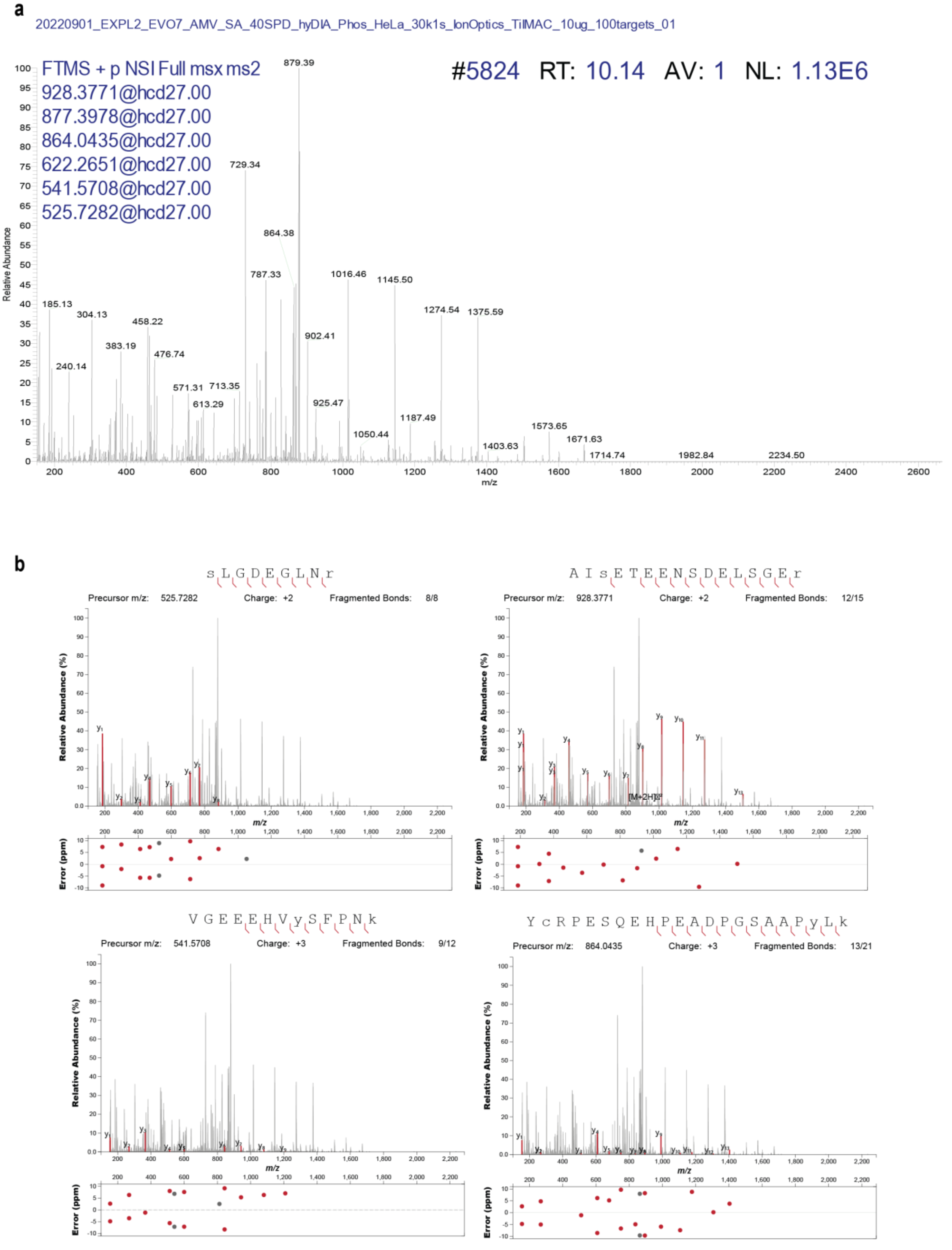

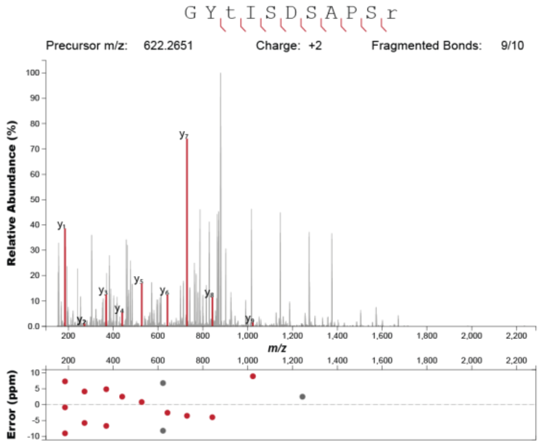

c

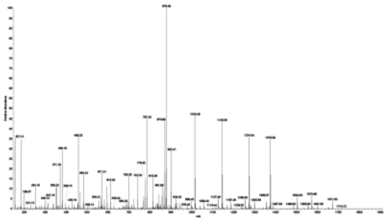

928.3771 and 923.3729

#5830

RT: 10.15

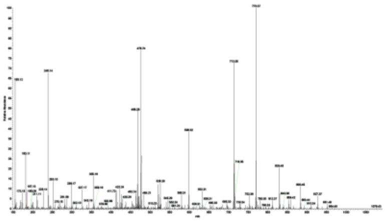

525.7282 and 520.7241

#5831

RT: 10.15

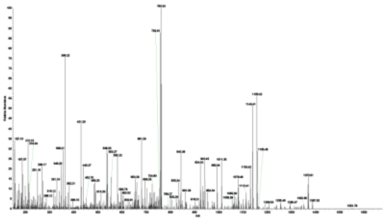

541.5708 and 538.8994

#5832

RT: 10.15

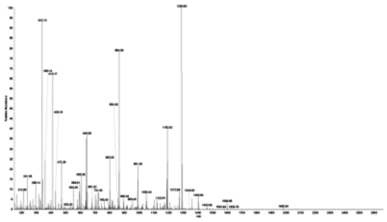

864.0435 and 861.3721

#5833

RT: 10.16

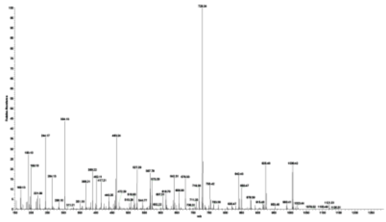

622.2651 and 617.2610

#5834

RT: 10.16

Supplementary Figure 1. Example of hybrid-DIA scans in one cycle. (A) Example of a multiplex-targeted MS2 scan combining six heavy labelled peptide standard found in the same full scan. (B) Fragment annotation for the five heavy labelled peptides identified by the hybrid-DIA API in the multiplex scan show in A. Annotation of the spectrum was performed using Interactive Peptide Spectral Annotator" from Brademan et al[1]. (C) Multiplex MS2 scans with the heavy and light peptides triggered by the hybrid-DIA API in the same acquisition cycle.

Supplementary Figure 2.

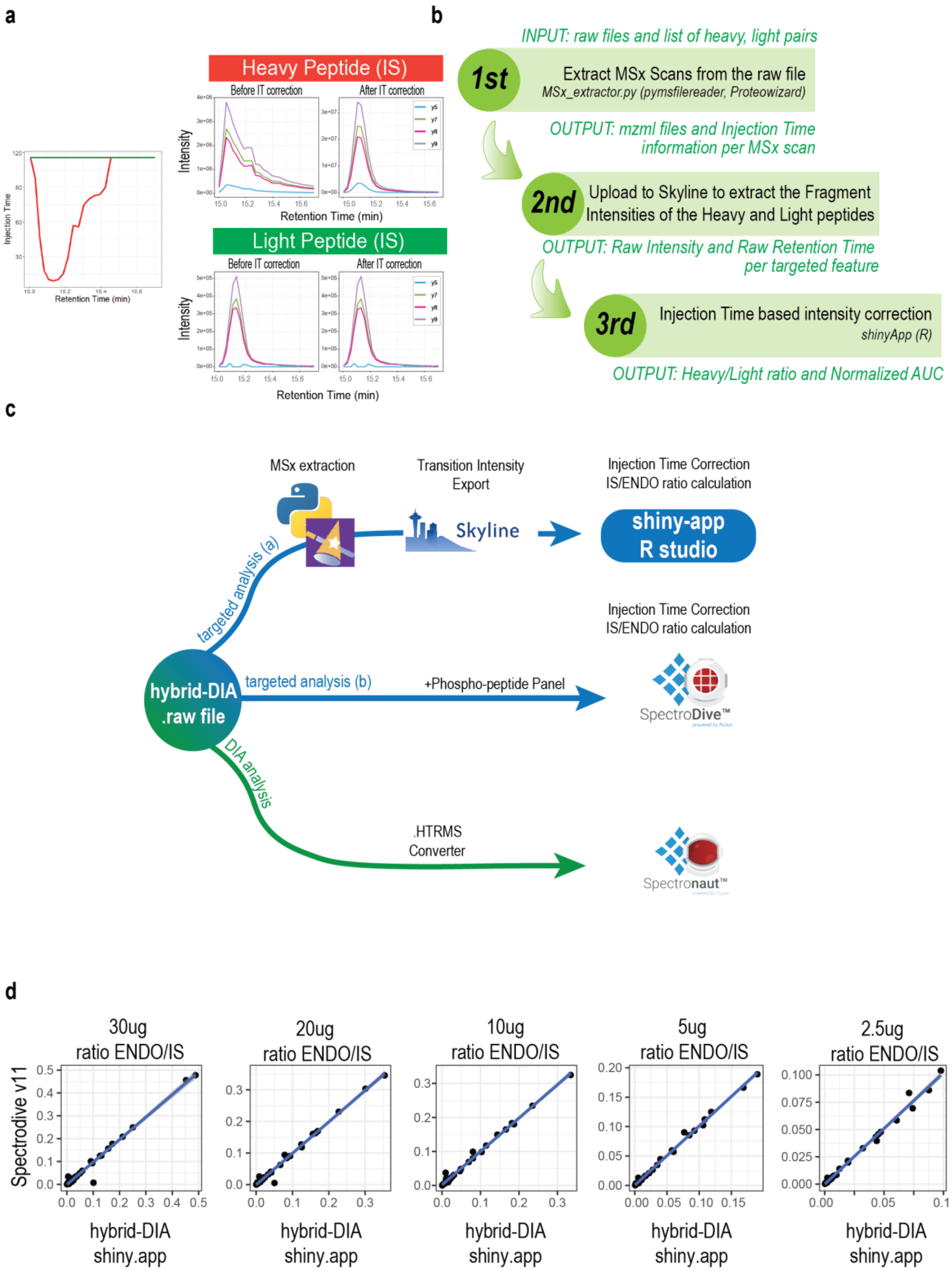

Supplementary Figure 2. Down-stream data analysis. (A) Example of the effect of the MS2 intensity profile due to the differential injection time for heavy and light peptides used in multiplexed scans and the result after correcting the MS2 intensities by that injection time. (B) Diagram of the three steps of the data analysis pipeline for extracting and processing the

targeted multiplexed scans generated with the hybrid-DIA method. (C) Alternatives for data processing of hybrid-DIA raw files. Targeted multiplex scans containing heavy-light (IS/ENDO) peptides can be processed with the hybrid-DIA pipeline described in Supplementary Note 2 (option a), or with the commercial software SpectroDive v11.1 (option b). DIA scans can be processed in Spectronaut prior to conversion to HTRMS format using the HTRMS coversor for hybrid-DIA raw files. (D) Plot showing the correlation between the quantitative results (expressed as ratio ENDO/IS) obtained when using SpectroDive v11.1 (q-value<=0.05) (x-axis) or the hybrid-DIA shiny-app pipeline (y-axis).

Supplementary Figure 3.

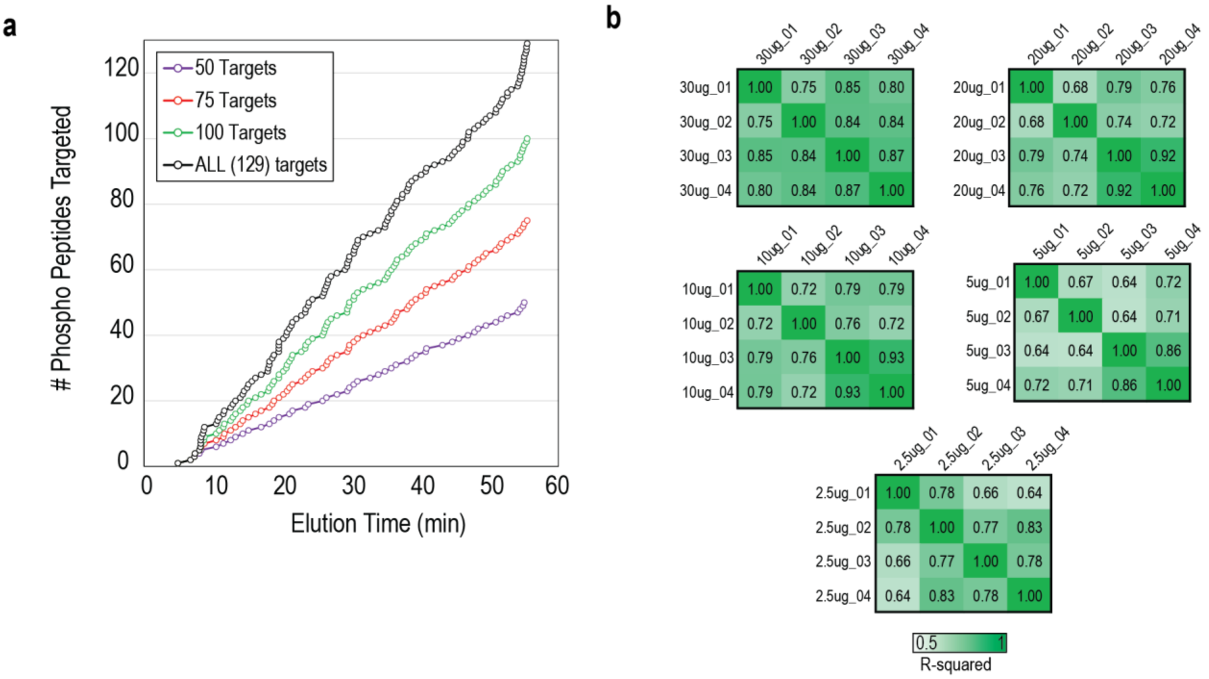

Supplementary Figure 3. Considerations for DIA analysis in hybrid-DIA runs. (A) Graph representing the distribution of the phospho-peptides from the SureQuant Multipathway Phosphorylation Kit (black), and the different subset of targets used for evaluating cycle time use: 50 (purple), 75 (red) and 100 (green). (B) Sample to sample correlation between DIA runs, measured as R-squared.

Supplementary Figure 4.

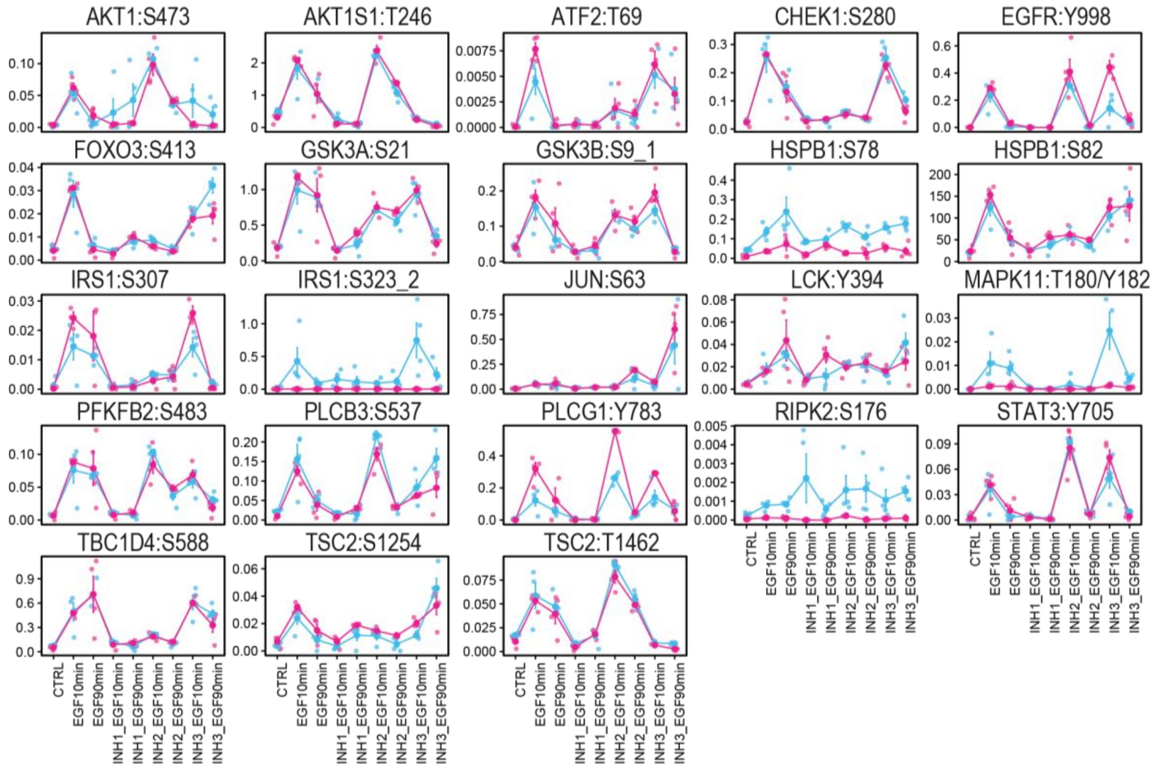

Supplementary Figure 4. Comparison of hybrid-DIA versus SureQuant methods to describe EGF signaling. Profile plot of regulated sites by EGF (two sided, two-sample t-test, log2 fold change (10 min vs control) >1.5, q-value<0.01). Dots indicate the absolute ratio Endogenous to Heavy standard. In blue, data from hybrid-DIA quantification (n=4 biological replicates); and in pink, data from SureQuant quantification (n=4 biological replicates). Line indicate the average of the experimental replicates, and the error bars the standard error of the mean.

Supplementary Figure 5.

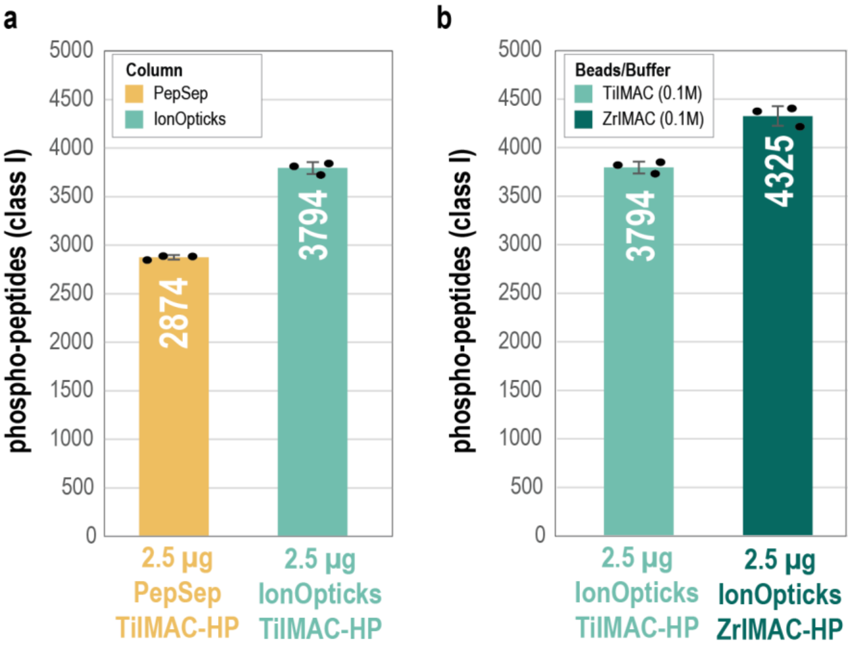

Supplementary Figure 5. Optimization of phospho-enrichment method for low input. (A) Comparative bar plot assessing the effect of the column type (PepSep vs IonOpticks) on the number of phospho-peptides (class I) identified when using 2.5 µg of peptide input for phospho-enrichment with TiIMAC-HP beads (n=3 experimental replicates). (B) Comparative bar plot assessing the effect of the beads type (TiIMAC-HP vs ZrIMAC-HP) on the number of phospho-peptides (class I) identified when using 2.5 µg of peptide input for phospho-enrichment with IonOpticks column (n=3 experimental replicates). Length of the bar indicates the average of the experimental replicates, and error bar the standard deviation. Source data are provided as a Source Data file.

Supplementary Figure 6.

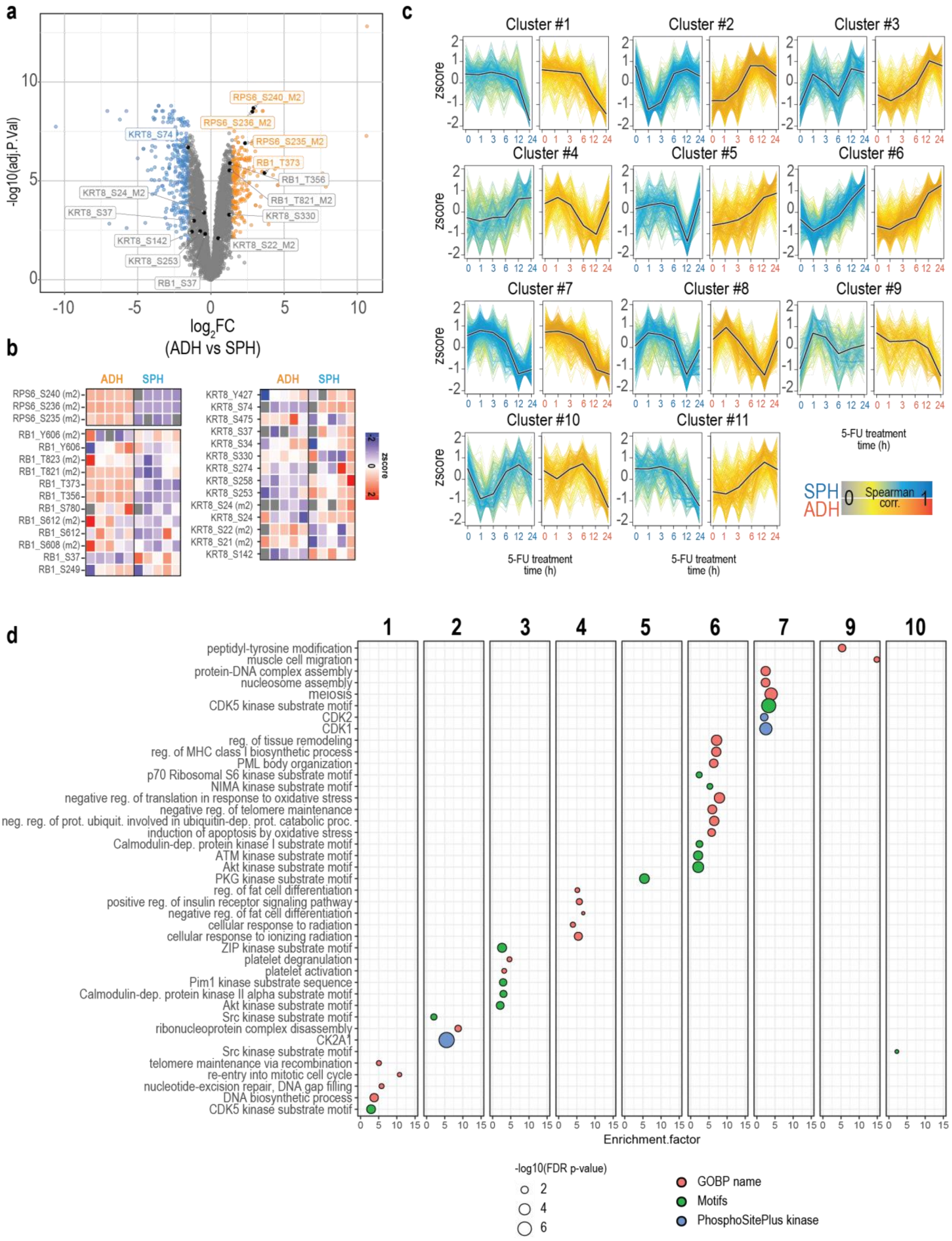

Supplementary Figure 6. Functional phosphoproteomics analysis of the response to 5-FU in different cell culture types. (A) Volcano plot comparing basal state (time zero of treatment) of HCT116 adherent cells (orange) and single multicellular spheroid (blue) (n=5 biological replicates, limma two-sample t-test, Benjamini Hochberg FDR). Labelled points

indicate representative proteins for each type of cell culture. (B) Heatmap of relative intensities of representative protein/phospho-site markers of monolayer and spheroid cell culture, highlighted in panel a. (C) Temporal profile clusters for spheroid (blue) and adherent cells (yellow) upon 5-fluorouracil treatment. Y-axis represents z-score scaled log2 phospho-site intensity. Dark line indicates the centroid of the cluster, and each line represents a different phospho-site. Color of the lines reflects the Spearman correlation to the centroid of the cluster. (D) Fisher's exact test (two-sided, Benjamini-Hochberg FDR) to evaluate enrichment of Gene Ontology Biological Process (GO-BP) terms, kinase motifs and PhosphoSitePlus kinases in the clusters defined in panel c. Only clusters with significant GO-BP terms are used in this panel.

Supplementary References

1. Brademan, D. R., Riley, N. M., Kwiecien, N. W. & Coon, J. J. Interactive Peptide Spectral Annotator: A Versatile Web-based Tool for Proteomic Applications. *Mol. Cell. Proteomics* **18**, S193–S201 (2019).
