# Supplementary material for: Hybrid-DIA: intelligent data acquisition integrates targeted and discovery proteomics to analyze phospho-signaling in single spheroids
Source: Nat Commun. 2023 Jun 16;14:3599. doi: 10.1038/s41467-023-39347-y (PMC10276052; doi:10.1038/s41467-023-39347-y)
Supplement: Supplementary file 3 — Description of Additional Supplementary Files Document [file 41467_2023_39347_MOESM3_ESM.docx]

**Description of Additional Supplementary Information Files**

**Supplementary Data 1.** Quantitative results of the targeted analysis of EGF stimulated HeLa cells in

the presence of three kinase inhibitors, either with hybrid-DIA or with SureQuant.

(A) Quantification for hybrid-DIA data. Data is provided as Area Under the Curve (AUC) of the

endogenous peptide. Hybrid-DIA data was normalized by the intensity of the heavy labeled standard

and missing values were imputed using values from a left-censored distribution, accompanied with

the statistical analysis (two-samples two-sided t-test, FDR corrected, 3 valid values in one

experimental group were required).

(B) Quantification for SureQuant data. Data is provided as ratio of the endogenous peptide versus the

heavy-labeled standard obtained from Skyline.

**Supplementary Data 2**. Quantitative results of the discovery analysis of EGF stimulated HeLa cells in

the presence of three kinase inhibitors, with hybrid-DIA and analyzed in Spectronaut (v17). Data has

been collapsed to phospho-sites, log2 transformed, normalized and imputed for missing values.

**Supplementary Data 3.** Quantitative results of the targeted analysis of colorectal cancer cells grown

in monolayer (adherent) or in 3D format (spheroid) treatment for 0, 1, 3, 6, 12 and 24 hours with 5-

fluorouracil.

(A) Quantification is provided as Area Under the Curve (AUC) of the endogenous peptide for adherent

cells.

(B) Quantification is provided as Area Under the Curve (AUC) of the endogenous peptide for single

spheroids.

(C) Statistical analysis results for adherent cells (two-samples two-sided t-test, FDR corrected, 3 valid

values in one experimental group were required). All statistical comparisons were performed using

time 0 as reference.

(D) Statistical analysis results for spheroids (two-samples two-sided t-test, FDR corrected, 3 valid

values in one experimental group were required). All statistical comparisons were performed using

time 0 as reference.

**Supplementary Data 4.** Differential regulation results of the discovery analysis of colorectal cancer

cells grown as spheroids (A) or as monolayer (B) after treatment for 0, 1, 3, 6, 12 and 24 hours with

5-fluorouracil. Data was obtained using Spectronaut (v17). Data has been collapsed to phospho-sites,

log2 transformed, normalized and imputed for missing values. Statistical analysis was performed using

limma robust t-test (two-samples, two-sided, FDR corrected).
